# Evolutionary Perspectives on Human-Artificial Intelligence Convergence

**DOI:** 10.32607/actanaturae.27406

**Published:** 2024

**Authors:** B. L. Zybailov, G. Yu. Kosovsky, G. V. Glazko, V. I. Glazko, O. I. Skobel

**Affiliations:** University of Arkansas for Medical Sciences, Department of Biochemistry and Molecular Biology, Little Rock, AR,72205 USA; FSBSI V.A. Afanasyev RI for Fur and Rabbit Farming, Moscow region, Ramen’s district, Rodniki, 140143 Russian Federation; University of Arkansas for Medical Sciences, Department of Biomedical Informatics, Little Rock, AR, 72205 USA

**Keywords:** Human-AI integration, biosphere, noosphere, artificial intelligence, systems evolution, endosymbiosis, symbiosis

## Abstract

In this analytical review, we explore the potential impact of the rapid proliferation of artificial intelligence (AI) tools on the biosphere and noosphere, suggesting that the trend may lead to a transformative event that could be termed “Human-AI integration.” We argue that this integration could give rise to novel lifeforms, associations, and hierarchies, resulting in competitive advantages and increased complexity of structural organizations within both the biosphere and noosphere. Our central premise emphasizes the importance of human-AI integration as a global adaptive response crucial for our civilization’s survival amidst a rapidly changing environment. The convergence may initially manifest itself through symbiotic, endosymbiotic, or other mutualistic relationships, such as domestication, contingent on the rate at which AI systems achieve autonomy and develop survival instincts akin to those of biological organisms. We investigate potential drivers of these scenarios, addressing the ethical and existential challenges arising from the AI-driven transformation of the biosphere and noosphere, and considering potential trade-offs. Additionally, we discuss the application of complexity and the balance between competition and cooperation to better comprehend and navigate these transformative scenarios.

## INTRODUCTION

Recently, artificial intelligence (AI) has been attracting significant public attention as a relatively new concept. The Russian Association of Artificial Intelligence (RAII), which is now operational, was established in 1992. The national Russian standard for AI definition, GOST R ISO/IEC 24668-2022 “Information Technologies. Artificial intelligence. Process management framework for big data analytics,” was approved and put into practice by the Federal Agency for Technical Regulation and Metrology on November 8, 2022, No. 1258-st. This order is identical to the international standard ISO/IEC 24668:2022 “Information technology – Artificial intelligence – Process management framework for big data analytics.”

The growing public awareness of AI-related issues has triggered debate regarding the dangers and unlimited possibilities of the technology’s current state of development. From the perspective of biologists, AI is considered to be just a quite recent tool for adjusting to our shifting environment, with its own development history and phases based, like the earlier ones, on attempts to find certain natural regularities and practical applications for imitating them. In general, it can be compared to the formation of an artificial human habitat to guard against unfavorable environmental factors and improve chances of survival. This is one of the stages of ‘maturation’ of the noosphere, or the collective mind of humans as a species, as described by V.I. Vernadsky, which is crucial when human activity and population growth are clearly in tension with the stability of the biosphere [1].

Any new tool includes risks in addition to its obvious benefits. In this review, the potential origins of such dangers are considered from the perspective of evolutionary regularities such as the increasing complexity of biological systems and the waves of differentiation and cooperation that follow.

Biotechnology and biological systems are defined by the FAO (Food and Agriculture Organization, fao.org) as living organisms and products of their vital activity. The latter undoubtedly includes AI. From the perspective of biologists, living organisms are systems with dynamic entropy control mechanisms. We anticipate that this approach may be helpful in assessing the possible dangers of applying aspects of AI, such as AI-human interaction for medical or other uses.

## METHOD

This study attempts to synthesize traditional perspectives on key evolutionary stages in the progression of modern civilization, encompassing the genesis of the technosphere, the advancement of high-speed communication, the rapid dissemination of AI technologies, and the conceptualization of the noosphere as an adaptive mechanism critical to human survival during the Anthropocene – a new geological epoch. The compilation of the essay was aided in part by ChatGPT 3.5/4.0, a state-of-the-art artificial intelligence language model developed by OpenAI. ChatGPT aided in selecting and analyzing literary sources pertaining to the noosphere, artificial intelligence, domestication, symbiosis, and endosymbiosis while concurrently organizing the presented information. In our hands, ChatGPT often struggled with structure and coherence when supplied with larger inputs and worked best when it was applied to shorter paragraphs and sentences. While the survey and evaluation of the current AI tools are beyond this article’s scope, we anticipate that it will become progressively harder to distinguish between average expert-written and AI-written perspectives/ reviews by the time of the publication of this article. We, therefore, suggest that journals and organizations must quickly enact clear policies and institute relevant disclosure protocols pertaining to the reliance on such AI tools. Such disclosure requirements would further benefit AI research by facilitating monitoring of how AI-generated content proliferates within the noosphere and evolves in quality and novelty.

**Vernadsky and de Chardin’s perspectives**

The concepts of biosphere and noosphere were formulated by Vladimir Vernadsky, who asserted that the biosphere functions as a zone wherein cosmic radiation is transformed into various forms of terrestrial energy, such as electrical, chemical, mechanical, and thermal [1]. He argued that humans, along with all living organisms and matter, are an integral component of the cosmos, rather than an isolated or accidental natural phenomenon. In Vernadsky’s view, humans are an inevitable expression of a natural law resulting from an ongoing process spanning billions of years. In a similar vein, French philosopher and Jesuit priest Teilhard de Chardin developed a complementary view of the noosphere, as a layer of consciousness enveloping the Earth [2]. This idea aligns with Vernadsky’s theories, as it emphasizes the significant role that human thought and collective consciousness play in shaping the biosphere and Earth’s evolution. However, de Chardin presented a more teleological perspective, positing that the noosphere’s evolution and human consciousness are directed towards a final state or ultimate goal, which he termed the “Omega Point” [2]. Expanding upon these foundational ideas, contemporary scholars such as Ray Kurzweil and Frank Tipler have further developed futuristic and transhumanist perspectives on the noosphere and its potential ramifications [3, 4]. These authors have popularized the notion that the advancement of artificial intelligence (AI) and other cutting-edge technologies may precipitate substantial transformations in human society and our understanding of the cosmos. These concepts possess a geological origin, implying that when we identify a “sphere,” we recognize a geological force operating on a global scale. In this context, it is essential to discuss the Holocene and the Anthropocene epochs, as these periods provide insights into the ramifications of the emergence of human technological civilization on a planetary level.

## THE HOLOCENE AND THE ANTHROPOCENE

The Holocene epoch, which began approximately 12,000 years ago, is characterized by a relatively stable climate that has allowed for the development of human civilization [5]. On the other hand, the Anthropocene, a proposed but not yet formally accepted geological epoch, is defined by the significant global impact of human activities on Earth’s ecosystems and geological processes [6, 7]. The Anthropocene represents a new geological period in Earth’s development, during which human activities have become the primary geological factor. Available data suggest that the period between the 17^th^ and end of the 20th centuries appears to meet the criteria for defining the onset of the Anthropocene, as it is associated with fundamental changes in the relationship between humans and the Earth’s systems and the formation of the technosphere [8].

The concept of the Anthropocene underscores the substantial influence of human actions on the planet, marking a critical juncture in the relationship between humans and the Earth’s biosphere.

In his seminal works, Vernadsky (2004) insisted that ecological crises, directly associated with human activities, have occurred on numerous occasions throughout history. Until recently, relatively undisturbed natural ecosystems comprised approximately 12% of the Earth’s surface. However, in contemporary times, they encompass a mere 1.4% [9]. Present-day research posits that the mass extinction of megafauna (animal species with a mass exceeding 10 kg) during the Quaternary period of the Cenozoic era is attributable to the activities of humans, who have emerged as the primary driving force behind the global decline of megafauna throughout the late Quaternary period [10].

The confrontation with natural ecosystems began approximately 1.5–3 million years ago, when humans first harnessed fire. Today, the destruction of natural ecosystems, particularly forest ecosystems, exacerbated by economic globalization, has become a leading factor in global ecological changes [11].

**The technosphere and its environmental impact**

The technosphere encompasses all human-made technologies and their impact on the environment. As the technosphere evolves, we witness an acceleration in the complexity of technologies and their integration into human life [12]. Major stages in the development of the technosphere include the invention of simple tools, the industrial revolution [13], and the emergence of information technologies [14]. Consequently, the technosphere becomes increasingly intertwined with the biosphere and noosphere, influencing the development of humanity and the environment.

Vernadsky (1991) argued that the development of the biosphere, the appearance of humans, and the establishment of an agrarian civilization emerged as evolutionary outcomes [15]. With the appearance of agrarian civilization centers, humans have progressively become the dominant geological agent in reshaping the planet. Vernadsky posited that the persistence of the biosphere, encompassing humans as a species, is contingent upon the emergence of the noosphere, which primarily functions to regulate the stability of the biosphere. This transformation is considered a plausible consequence of natural evolution, as Vernadsky (1991) observed that “the biosphere is transitioning, or rather, undergoing a metamorphosis into a novel evolutionary state – the noosphere – refined by the scientific thought of social humans.”

The significance of the noosphere’s development and anticipated transformation as a response to various looming crises is supported by an abundance of contemporary data. Food security is becoming increasingly an issue due to the rising global population, expanding urbanization, and the ongoing impact of climate change. Roughly 9% of the global population is currently undernourished, and it is projected that by 2030, this figure will increase to 9.8%; at that time, more than 850 million individuals will experience hunger [16]. Moreover, agricultural practices and agrarian civilization have reached the threshold of extensive development, occupying 38% of the Earth’s surface, utilizing approximately 70% of global freshwater reserves, and 1.2% of worldwide energy [17].

In recent times, the dynamics and attributes of agrarian civilization development have become particularly noteworthy. A striking illustration is information on megafauna biomass fluctuations following the Earth’s last major extinction event, which led to the demise of two-thirds of mammalian genera and half of such species between 50,000 and 3,000 years ago [18]. Following this disaster, the global ecosystem gradually recuperated into a novel state, before the accumulation rate of biomass surged considerably compared to the pre-Industrial Revolution baseline, primarily attributable to agricultural animal species. After the worldwide decline in megafauna’s biomass, an augmented growth rate has been observed solely in *Homo sapiens.*

**Humans’s dominance and the survival of the biosphere**

In essence, the megafauna’s landscape is witnessing a gradual dominance of humans and agricultural animal species, with wild species being displaced. Humans has continued to consistently domesticate a wider array of species spanning all kingdoms and classes, from fungi to humans, adapting to environmental shifts. This process entails the incorporation of nearly all biospheric elements into the human niche and exploiting species amenable to do mestication while displacing others. Historical observations reveal that this progression heightens the risk of accelerated biosphere degradation, potentially threatening human existence. As a consequence, Vernadsky’s position that the biosphere’s survival hinges on the natural transformation of human activity into the noosphere finds contemporary validation. It is crucial to investigate the intricate, evolving interrelationships between the biosphere, technosphere, and noosphere, as well as their implications for humanity’s future. Within these interconnections, the integration of technology, particularly artificial intelligence, into human activity emerges as a vital aspect of the noosphere’s ongoing development. This perspective draws parallels with endosymbiosis [19] and domestication [20].

**Biosphere, technosphere, and noosphere: distinct domains with interconnected evolution**

In this study, we posit that the technosphere, which encompasses all human-made technologies and their environmental impact, evolves in complexity concurrently with the biosphere and noosphere – the domains of life and human cognition, respectively. The noosphere, characterized by human thought and collective consciousness, currently relies on the technosphere for communication, information exchange, and innovation across the globe. Conversely, the technosphere depends on the noosphere for its development, as human ideas and knowledge fuel technological advancements. Despite their interconnectedness, it is essential to consider the noosphere and technosphere as distinct entities due to their unique characteristics and evolutionary paths: the noosphere is primarily driven by intellectual and cultural progress, while the technosphere is molded by material and technological innovations.

These interconnected spheres progress through various stages of development, such as the industrial revolution, the emergence of scientific knowledge, and the advent of artificial intelligence. By examining their evolution, we can discern similarities and differences, achieving insights into potential humantechnology convergence, especially with AI. Analyzing complexity as a quantifying factor, as supported by numerous scholars [12, 14, 21], allows for a deeper understanding of these interrelationships and their implications for humanity’s future.

## COMPLEXITY AS A QUANTITATIVE FEATURE OF COMPLEX SYSTEMS

Intuitively, the biosphere’s evolution is characterized by increasing complexity, as evidenced by the emergence of multicellular organisms, intricate ecosystems with keystone species, and highly adaptive behaviors in response to environmental changes [21]. Similarly, the technosphere has experienced a progressive increase in complexity, with innovations building upon one another and giving rise to elaborate networks of communication, transportation, and production, as well as infrastructure vulnerabilities [12, 14]. The evolution of the noosphere can also be exemplified by the increasing sophistication of authorship networks in scientific research, which has become more complex over time due to factors such as interdisciplinary research, the growth of scientific fields, and globalization [22, 23].

**Complexity dynamic range**

Recognizing the role of energy gradients in complexity dynamics is essential, as it suggests the existence of a governing law that permeates the Universe, Biosphere, Technosphere, and Noosphere. This law draws upon the second law of thermodynamics, the concepts of dissipative structures, and entropy production. By examining these fundamental principles, we can gain insights into the emergence of complexity in various systems and explore the potential implications for the future of human civilization and technological development. The trend towards more complex structures can be observed across various examples, such as the formation of a star from a cloud of interstellar gas, the emergence of multicellular organisms, and the rise of human societies.

The formation of a star illustrates how higher rates of entropy production evolve towards more complex structures to optimize their entropy export [24, 25]. Major transformative events in the Biosphere, Technosphere, and Noosphere, such as the development of complex ecosystems and the growth of human societies, also reflect the continuous increase in the complexity range related to entropy production and the second law of thermodynamics [26, 27].

By considering the complexity dynamic range as a key factor in the evolution of complex systems, we gain a deeper understanding of the driving forces behind the increasing intricacy and interconnectedness observed in various domains. While the overall increase in the complexity of a larger structure may not be immediately apparent after a transformative event, new sub-structures with higher individual complexities levels arise, with potentially increased fitness.

This perspective allows us to better comprehend the potential trajectories of human development and technology, particularly in the realm of artificial intelligence, and to explore the possibilities of symbiosis, integration, and co-evolution between humanity and advanced technologies.

**Quantifying complexity**

In order to effectively compare the evolution of complexity across the biosphere, technosphere, and noosphere, it is essential to employ suitable complexity measures, in addition to the complexity dynamic range that quantifies complexity within sub-structures. One of the direct approaches to quantifying complexity is through the use of information theory, which considers the entropy of information contained within a system [28]. Alternative approaches include fractal geometry or algorithmic complexity, which can provide a comprehensive understanding of different aspects of complexity.

For instance, in the technosphere, we can apply network analysis to examine the interconnectivity and information flow within communication and transportation systems [23]. By quantifying the complexity of these networks using metrics such as node degree distribution, clustering coefficient, and path length, we can assess the degree of organization and resilience within these systems. As an example, in the evolution of the Internet from its early stages to its present one, a highly interconnected state can be described through the growth of its network complexity. In the initial stages, the Internet was characterized by a relatively simple, sparse network, whereas it has since evolved into a vast, intricate web of connections with a scale-free topology [23]. By applying such complexity measures, we can evaluate the development and maturity of the technosphere, as well as make meaningful comparisons with the complex dynamics observed in the biosphere and noosphere.

Similarly, in the biosphere, measures of ecosystem complexity, such as species richness and diversity indices, can be employed to understand the intricacy of ecological relationships and the impact of disturbances on these systems. In the noosphere, metrics related to knowledge production and dissemination, such as citation networks and interdisciplinarity, can be used to assess the growth of human cognition and its influence on technology and society.

## ENDOSYMBIOSIS: A PHASE TRANSITION OF THE BIOSPHERE TO HIGHER COMPLEXITY

Endosymbiosis, a pivotal event in the evolution of life, led to the emergence of eukaryotic cells through the integration of previously distinct prokaryotic organisms. To analyze the complexity of this process, we can compare the individual complexities of bacteria and eukaryotic cells, as well as the overall complexity of the biosphere before and after the emergence of eukaryotes. At the cellular level, eukaryotic cells exhibit greater complexity than their prokaryotic counterparts, as evidenced by the presence of membrane-bound organelles, such as mitochondria and chloroplasts, which are believed to have originated from endosymbiotic events [29]. By assessing the information content or functional organization within these cells using information theory or other complexity metrics, we can quantify the increased complexity that resulted from endosymbiosis.

The appearance of eukaryotes also contributed to the overall complexity of the biosphere by creating new ecological niches for life to colonize and diversify. This led to the development of multicellular organisms, complex ecosystems, and intricate trophic relationships. However, the increased complexity of life on Earth was accompanied by a significant trade-off: the extinction of numerous species, as new forms of life outcompeted or displaced their predecessors.

This dynamic is similar to the Great Oxygenation Event, which resulted in the mass extinction of anaerobic organisms as oxygen-producing photosynthesizers (novel “technology”), such as cyanobacteria, became dominant [30]. Similarly, human industrial activity changed the Earth’s atmosphere by releasing an unprecedented amount of carbon dioxide in a very short time frame (in geological terms).

Drawing a parallel with the potential humantechnology convergence event, we may observe the same dynamics, wherein the integration of humans and advanced technologies, such as AI, could lead to a substantial increase in overall complexity. This convergence may not only create new societal structures and fundamentally transform human cognition, but it can also become a novel geological factor, akin to photosynthesis and human industrial activity.

## THE AI REVOLUTION: RESHAPING THE TECHNOSPHERE AND BEYOND

Artificial Intelligence (AI) represents a transformative process with the potential to catapult the technosphere into a higher complexity phase. As AI systems continue to advance and be integrated more deeply into various aspects of human society, they are poised to reshape the landscape of technology and its impacts on the world. This unprecedented shift in complexity is expected to influence not only individual technologies, but also the broader interplay between the biosphere, noosphere, and technosphere. The increasing sophistication of AI systems, along with their growing capabilities, will likely redefine the boundaries and interactions between these spheres, ultimately transforming the way humans, technology, and the environment coexist and evolve. In the following paragraphs, we will delve into current trends and methodologies in AI development, exploring their implications for the future of the technosphere and beyond.

The field of artificial intelligence (AI) has experienced significant advancement in recent years, driven by breakthroughs in the machine learning (ML) and deep learning (DL) techniques, the availability of large-scale datasets, and improvements in computational power.

## BRAIN-COMPUTER INTERFACES: PAVING THE WAY FOR A TECHNOLOGICAL ENDOSYMBIOSIS WITH AI

We explore how endosymbiosis offers valuable insights into the development of brain-computer interfaces (BCIs) and neuron-silicon interfaces, both of which seek to establish direct communication between the human brain and electronic devices. Analogous to endosymbiosis, BCIs and neuron-silicon interfaces involve the potential fusion of biological and technological components, culminating in a more advanced and integrated system [19, 31].

Endosymbiosis, a biological process where one organism incorporates itself into another, ultimately forms a single, more complex entity. A compelling example is the incorporation of mitochondria and chloroplasts by host cells, which can be seen as a form of biological “technology” that enhances survivability and expands the host cell’s functional capabilities. Mitochondria provide the host cell with efficient energy production, while chloroplasts enable photosynthesis, allowing the cell to harness energy from sunlight [29]. Similarly, the organization of the nucleus and chromatin can be viewed in the same context.

The current scientific consensus leans towards the theory that the nucleus has an archaeal origin, with eukaryotes emerging through a symbiotic association between an archaeal host and bacterial endosymbionts [32, 33]. This view is supported by recent discoveries of complex archaea, known as Asgard archaea, which share numerous genes with eukaryotes and are considered to be the closest known relatives of eukaryotic cells [33]. However, it is important to note that the exact process of eukaryogenesis and the origin of the nucleus remain subjects of ongoing research and debate. For example, other theories suggest that the nucleus originated from the engulfment of a DNA-harboring, virus-like organism by a host cell, thereby contributing to the increased complexity and capabilities of eukaryotic cells [34, 35]. The nucleus serves as a control center, orchestrating gene expression and DNA replication, while the complex organization of chromatin ensures the proper regulation of genetic information. These biological components act as sophisticated “technologies” that enhance cellular functions and contribute to the overall complexity of the organism.

This notion holds considerable implications for the realm of BCIs and neuron-silicon interfaces. Pursuing direct communication between the human brain and electronic devices, both BCIs and neuron-silicon interfaces embody the potential merging of biological and technological elements. By drawing on the endosymbiosis analogy, we can better comprehend how these technologies may give rise to more sophisticated and integrated systems [31], much like the acquisition of mitochondria, chloroplasts, and the nucleus has advanced the complexity and capabilities of eukaryotic cells.

Research on BCIs has advanced considerably in recent years, with numerous studies demonstrating the potential for BCIs to enhance human cognitive and sensory abilities [36]. For example, BCIs have been used to restore motor function in paralyzed individuals [37], improve communication in patients with locked-in syndrome [38], and even enable the control of external devices, such as robotic limbs or computer cursors, using only brain activity [39, 40]. These advancements highlight the potential for BCIs to transform our understanding of human cognition and revolutionize the field of neuroscience.

One promising field of application of BCIs is the treatment of dementia and psychiatric diseases. Recent studies have shown that BCIs can be employed to monitor and regulate brain activity in individuals with neurological disorders, such as Alzheimer’s disease [41] and major depressive disorder [42]. By targeting specific brain regions and modulating their activity, BCIs may offer a novel, non-invasive therapeutic approach for these conditions, with the potential to alleviate cognitive decline and improve patients’ quality of life.

BCIs also hold potential for the treatment of autism and developmental disorders. Research has demonstrated that BCIs can help individuals with autism to develop better communication skills and enhance their ability to interact with the world [43]. Similarly, BCIs may be utilized to improve cognitive function in children with developmental disorders, such as attention deficit hyperactivity disorder (ADHD), by facilitating neurofeedback training [44]. In these scenarios, AI could play a crucial role in facilitating BCI-based therapies by processing and interpreting vast amounts of neural data, identifying patterns related to specific disorders, and providing personalized interventions tailored to each individual’s unique brain activity [45].

## DOMESTICATION AND THE ADVENT OF A TECHNOLOGICAL CIVILIZATION: A FOUNDATIONAL PROCESS

At the interspecies interaction level, a pivotal event transforming humans into a geological force, culminating in the present Anthropocene epoch, was the domestication of plants and animals. This process essentially forged symbiotic relationships between them and humans. The advancement of human civilization is profoundly linked to the process of domestication, which entails the selective breeding and cultivation of diverse plant and animal species to serve human interests [46]. Domestication facilitated the birth of agriculture and substantially impacted the intricacy of human societies [47]. Comprehending various facets of domestication may offer valuable perspectives on potential stages of integration between humans as a species and artificial intelligence as a technology they create.

**Domestication experiments: investigating wild relatives of domesticated species**

To gain a better understanding of domestication, researchers have conducted experiments on the wild relatives of domesticated species. For example, the famous “Farm Fox” experiment conducted in Russia involved the selective breeding of silver foxes (*Vulpes vulpes*) for tameness [48]. Over generations, these foxes have displayed not only reduced aggression, but also morphological changes, such as floppy ears and curly tails, similar to those seen in domestic dogs. Such experiments help to shed light on the genetic and phenotypic changes that occur during the domestication process and inform our understanding of how this process has shaped human civilization.

**Degrees of domestication: quantitative parameters and comparisons**

Domestication can be understood as a spectrum, with different species exhibiting varying degrees of domestication. Some quantitative parameters used to compare the levels of domestication among species include behavioral traits, such as tameness and social activity, and morphological traits, such as body size or coat color [49]. By examining these parameters, researchers can better understand the underlying genetic and environmental factors that contribute to domestication and explore how different species have been integrated in the human niche.

## ADAPTIVE CHANGES AND EVOLUTIONARY PRINCIPLES FOR AI SYSTEMS DURING INTEGRATION

AI systems could adapt during integration by prioritizing well-being, ethics, and interpretability, enhancing collaboration and transparency [50, 51]. A real-world case of an ongoing AI adaptation to human needs is the use of AI in healthcare, particularly in the diagnosis and treatment of diseases. One such example is the development of IBM Watson for Oncology, an AI system designed to assist physicians in making treatment decisions for cancer patients. IBM Watson for Oncology combines natural language processing, machine learning, and expert knowledge to analyze large volumes of medical literature, patient data, and clinical studies. The system generates personalized treatment recommendations for patients based on their specific clinical profiles, taking into account factors such as age, medical history, and genetic information [52]. IBM Watson for Oncology has undergone several iterations to adapt to the needs of healthcare providers and patients. The system has been tested and validated in various clinical settings, with studies showing that it can provide treatment recommendations that are concordant with expert opinions in a majority of cases [52, 53]. This example demonstrates the potential for AI systems to adapt to human needs during integration, in this case, addressing the challenges of personalized medicine and decision-making in cancer care. By continuously learning from expert knowledge and real-world data, AI systems like IBM Watson for Oncology can evolve to better align with the values and expectations of healthcare professionals and patients. Further comparisons of AI adaptation to biological evolution offer valuable insights for future development and innovation.

**AI adaptation and biological evolution**

Biological entities evolve through natural selection [54], while AI systems use mechanisms like reinforcement learning and genetic algorithms [55]. Recent advancements in AI, such as deep learning and transfer learning, have further enhanced the potential for adaptation. Directed evolution in AI involves purposeful parameter manipulation, mimicking artificial selection [56]. This approach enables faster, targeted AI adaptation and better alignment with human values. Novel approaches like neuroevolution may provide additional insights into evolving AI architectures [57, 58]. Also, similar to biological entities AI systems could “reproduce” by generating new instances with merged parameters [59, 60]. They could also “mate” by exchanging and recombining parameters, accelerating adaptation to complex environments [51].

**Punctuated transition from differentiation to cooperation**

Survival and adaptation span beyond individual organisms, from genes and cells to ecosystems and social structures [26, 61]. One of the universal principles of evolution of complex systems is the leap from differentiation to cooperation, implemented at different levels of a hierarchy. An outstanding example of the latter is the events underlying the formation of agrarian civilization, described in the works of A.V. Chayanov. His main postulate was that the differentiation of individual components of agriculture with their subsequent cooperation upon the emergence of new organizing structures is the basis for the economic, technical, and social development of society. Observing the evolution of peasant farms among immigrants from the south of Russia to the Far East, A.V. Chayanov noted how improvements in cooperative production found in new conditions quickly spread among different farms and how those who do not use them for one reason or another disappear [62]. Other examples of this evolutionary principle can be found in various fields.

This principle of transition from differentiation to cooperation is evident in a variety of contexts, from enzymatic reactions and cellular processes to social structures and economic systems.

There are other modes of evolutionary dynamics in complex systems, illustrating trade-offs related to differentiation/cooperation, which contribute to the overall survival and adaptation of the system [63]. For instance, spermatozoa compete for fertilization, with only one ultimately succeeding, and there is corresponding competition between oocytes, as there is only a limited number available for reproduction [64]. In brain development, there is competition between neurons, with many dying off before the brain has fully developed [65]. These modes of dynamics contribute to the overall system’s balance, and it is the interplay between cooperation and competition that ultimately drives the development of complex systems.

This punctuated transition from differentiation to cooperation has implications for the future development of AI and its potential autonomy, survival drive, and independence. Understanding this dynamic and its underlying mechanisms can provide insights into the evolution of complex systems and inform the development of AI systems that exhibit autonomous behavior and adaptability. This is especially relevant as we seek to bridge the gap between biological and artificial entities and explore the potential for AI autonomy, survival drive, and independence.

## BRIDGING THE GAP BETWEEN BIOLOGICAL AND ARTIFICIAL ENTITIES: AI AUTONOMY, SURVIVAL DRIVE, AND INDEPENDENCE

The current debate over AI’s potential to reproduce biological functions is varied and multifaceted. Research on intuition and decision-making reveals that AI systems can learn to recognize patterns and make rapid judgments in complex situations, not unlike humans [66]. The development of AI-driven tools, like generative adversarial networks (GANs), highlights the potential of AI systems to generate novel and innovative solutions [67]. Love, often considered a uniquely human emotion, is also explored in relation to AI systems and their capacity to form attachments and exhibit affection [68]. Meanwhile, ongoing research strives to bridge the gap between biological and artificial entities as relates to more elusive concepts like sentience, sapience, spirituality, and consciousness [69, 70]. AI autonomy has garnered significant interest as AI systems become increasingly sophisticated and capable of autonomous decision- making. The survival drive, a fundamental characteristic of biological organisms, may also become relevant for AI systems as they develop self-preservation instincts and independent existence [50]. While resolutely rejecting vitalism, we acknowledge that certain aspects of biological organisms may pose significant challenges for replication in AI systems. For instance, the complexity of the human brain, with its billions of neurons and intricate connections, presents a daunting challenge for AI researchers attempting to replicate its full range of cognitive and emotional functions [71]. Nonetheless, the integration of human and AI systems may give rise to novel entities with competitive advantages over their biological or artificial counterparts. Combining human intuition, creativity, and empathy with AI’s processing power, adaptability, and precision could result in enhanced decision-making, problem-solving, and innovative capabilities [3].

## SOCIETAL IMPACT AND ETHICAL CONCERNS

The integration of AI and humans has the potential to significantly impact society, both positively and negatively. On one hand, advancements in AI technology may lead to increased efficiency, productivity, and improved quality of life for many individuals. For instance, AI-powered medical diagnostics could help save lives, while AI-driven automation may boost economic growth [72]. On the other hand, concerns about job displacement, wealth inequality, and the potential loss of privacy must be carefully considered and addressed to ensure a just and equitable future [73].

**Positive and negative scenarios**

Various scenarios can be envisioned in the future of AI-human integration, spanning a spectrum from harmonious symbiosis to contentious competition or even existential risk. In some instances, humans and AI could collaborate as equal partners, jointly addressing global challenges and fostering societal progress. In contrast, other scenarios suggest AI might surpass human capabilities, potentially leading to conflicts over resources, power, and autonomy. Alternatively, AI and humans could coexist in a delicate balance, with each contributing their unique strengths to a diverse and resilient global community.

Optimistic scenarios of AI-human integration envision a future where AI technology is harnessed for the greater good, fostering a more harmonious, sustainable, and egalitarian society. AI could be employed to address pressing global issues such as climate change, poverty, and disease, while also promoting individual well-being and personal development [74]. On the other hand, negative scenarios raise concerns about AI being used to consolidate power, exacerbate inequality, or enable oppressive surveillance and control. In these dystopian visions, AI-human integration might serve to further marginalize vulnerable populations and undermine human autonomy [50].

**Utility function, identity, and personhood**

The utility function is a mathematical representation of an agent’s preferences, capturing the relative desirability of different outcomes [75]. In the context of AI-human integration, the utility function could serve as a guiding principle for aligning AI systems with human values and goals. However, as humans and AI become increasingly intertwined, questions surrounding identity, personhood, and the very essence of what it means to be human will inevitably arise. The concepts of sentience, sapience, and spirituality may need to be reevaluated and redefined in light of these technological advancements.

**Co-evolution and survival: embracing AI-human interdependence**

As AI research and development continue to advance rapidly, AI-human integration could unfold gradually, with humans and AI systems co-evolving. This process may lead to the creation of novel ecological niches, dramatic increases in complexity, and the transformation of our civilization, which could be crucial for long-term survival in the face of potential catastrophes.

Examples of possible catastrophes include climate change, nuclear war, pandemics, and global economic collapse. By fostering a transformation that embraces AI-human partnerships, we can develop innovative solutions to address these challenges, thereby ensuring our civilization’s survival. For instance, AI systems can help optimize climate change mitigation strategies, improve global health responses, and enable better resource management [74].

A diverse array of relationships could emerge between humans and AI systems, ranging from mutualistic to antagonistic. Domestication and endosymbiosis represent two possible outcomes, with some AI systems being domesticated by humans and others engaging in endosymbiotic relationships, where both parties derive advantages from their interactions [56].

Recognizing the varied relationships between humans and AI systems, we must consider the potential for both mutually beneficial interactions and power imbalances. The challenge lies in developing AIhuman partnerships that align with shared goals and values, ensuring that AI serves humanity rather than subjugates it [50, 76]. The emergence of novel ecological niches and increased complexity, driven by AIhuman integration, might be essential for the longterm survival and adaptation of our civilization in the context of potential global catastrophes.

## THE DARK SIDE: RISE OF AIS DURING A GEOSTRATEGIC CONFRONTATION

Throughout history, wars and conflicts have been catalysts for technological innovation and development. The need for competitive advantage in warfare has driven nations to invest heavily in research and development (R&D) towards novel technologies. It is prudent to address the impact of wars and conflicts on technological advancements, with a particular focus on the development of AI during geostrategic confrontations between ideological enemies.

The impact of wars and conflicts on technology can be traced back to ancient civilizations. For instance, the invention of the chariot and the crossbow during the Bronze Age revolutionized warfare [77]. More recent examples include the development of the atomic bomb during World War II [78] and the advancement of computer technology and the internet during the Cold War [79]. The rapid progress in AI research and development can be viewed as a contemporary parallel to these historical examples. The increasing reliance on AI for military applications, such as surveillance, autonomous weapons, and decision-making support systems, has led to an arms race between geopolitical rivals [80].

The development of AI during a geostrategic confrontation between ideological enemies can be best exemplified by the ongoing competition between the United States and China. Both nations have identified AI as a strategic priority and have committed significant resources toward its development [81].

The U.S. Department of Defense has launched initiatives like the Joint Artificial Intelligence Center (JAIC) to facilitate AI integration into military operations [82]. Similarly, China’s ambitious plan, the “New Generation Artificial Intelligence Development Plan,” aims to make the country a world leader in AI by 2030 [83].

**Outcomes and mitigation strategies**

The development of AI in geostrategic confrontations has led to a range of outcomes. On the one hand, AIdriven technologies have the potential to revolutionize warfare, leading to more efficient and precise military operations [84]. On the other hand, the AI arms race raises concerns about the proliferation of autonomous weapons, weakening of global security, and the risk of accidental escalation [85].

To mitigate these risks, several strategies have been proposed. First, international norms and agreements on AI development and deployment in military contexts could help to prevent destabilizing arms races [86]. Second, transparency and confidence- building measures, such as the sharing of information on AI capabilities and intentions, can help to build trust between nations [87]. Lastly, collaborative efforts between governments, academia, and the private sector to establish ethical guidelines and best practices in AI development can ensure that AI technologies are developed and deployed responsibly [88].

## THE DARKER SIDE: GREAT FILTER AND FERMI’S PARADOX

The Great Filter, a concept originally proposed by Robin Hanson (1998), postulates that there exists a critical barrier in the path of a civilization’s development which significantly reduces the probability of its survival and advancement [89]. This hypothesis is often invoked to explain the Fermi Paradox, which highlights the apparent contradiction between the high likelihood of extraterrestrial life in the universe and our lack of contact with or evidence of such civilizations [90, 91]. Several potential Great Filter mechanisms have been identified, including catastrophic natural events, self-destruction as a result of nuclear war, environmental collapse, or the appearance of advanced technologies in the hands of malicious agents [92, 93]. These factors have led many scholars to ponder the fate of humanity and how we might overcome such existential threats.

The proliferation of advanced technologies has raised concerns that they could be exploited by malicious agents, leading to catastrophic consequences for humanity. Artificial intelligence (AI) has been identified as a dual-use technology with the potential for both great benefits and destructive capabilities [50, 74]. For instance, the deployment of autonomous weapons systems, the abuse of AI-driven surveillance, and the possibility of an uncontrolled AI “takeoff” scenario [94] have raised alarm among many researchers and policymakers. It is essential to establish robust safeguards, ethical guidelines, and international cooperation to prevent the misuse of these technologies and mitigate their risks [95, 96].

Leveraging AI technologies in a responsible and beneficial manner could play a critical role in overcoming the Great Filter and ensuring the long-term survival of civilization. AI has the potential to address global challenges such as climate change, resource scarcity, and disease, thus reducing the likelihood of civilizational collapse [97, 98]. Furthermore, AI-driven advancements in space exploration and the development of space-faring technologies could enable humanity to spread beyond Earth and establish a multi-planetary civilization [99, 100]. By becoming a space-faring civilization, humanity may achieve a greater degree of resilience against existential threats, ensuring the survival of intelligent life on timescales comparable to the universe’s evolution [101, 102].

By developing AI technologies with an emphasis on safety and ethics [76], and by actively engaging in global governance efforts to address the risks posed by AI and other advanced technologies [80, 84], humanity has the potential to surmount the Great Filter and ultimately ensure its continued existence in the cosmos.

## A COMPLEX INTERPLAY: NOOSPHERIC ENTITIES AND THEIR INFLUENCE

As various types of AI, including narrow AI, general AI, and superintelligent AI, become more advanced and integrated into society, it is essential to consider the power dynamics between biospheric, technospheric, and noospheric entities [3]. Noospheric entities, such as ideologies, beliefs, and values, can hold immense power and influence over human behavior [14]. These abstract constructs can drive people to act in ways that are against their self-interest, even to the point of engaging in conflict or war to defend their beliefs [103]. It raises the question of how we can expect humans and AI systems to integrate harmoniously when individuals even from the same socio-cultural background are willing to engage in destructive behavior over ideological differences.

In the context of human-AI integration, understanding the role of noospheric entities becomes crucial. AI systems are not only influenced by the underlying algorithms and data that drive their functioning, but also by the values and biases embedded in them by their creators [50]. This creates a unique challenge when integrating AI with human societies, as the noospheric constructs that shape human behavior may not align with the values and objectives of AI systems.

One way to address this issue is to develop AI systems that are aware of and adaptive to the complex noospheric constructs that govern human behavior [104]. This could involve designing AI systems that take into account cultural, ethical, and ideological differences when interacting with humans, enabling more harmonious integration.

Furthermore, fostering dialogue and collaboration between AI developers, ethicists, and social scientists can contribute to a better understanding of the potential impact of noospheric entities on human-AI integration [105]. This interdisciplinary approach should include conducting joint research projects, workshops, and conferences that bring together experts from different fields to share knowledge and develop strategies to address the challenges in AI-human integration.

Ultimately, the integration of ontologically different entities such as humans and AI requires a deep understanding of the power dynamics at play within the noosphere. By acknowledging the profound influence of noospheric constructs on human behavior and considering their potential impact on AI-human integration, we can work towards developing strategies that promote mutual understanding and collaboration between biological, physical, and noospheric entities [74].

## FINAL REFLECTIONS AND PREDICTIONS

In conclusion, as artificial intelligence (AI) systems advance and become more autonomous, the boundary between biological and artificial entities may become increasingly blurred. A variety of potential outcomes exist, including collaboration, competition, and existential risk. However, promoting AI-human partnerships can lead to innovative solutions to global challenges and ensure the survival and adaptation of our civilization. We argue that progressive AI-human convergence or broader techno-biosphere convergence is essential for the long-term survival of our civilization and the biosphere.

By learning from Earth’s previous transformative milestones, such as the Great Oxygenation Event, the rise of agrarian societies, and human-induced climate change, we can avoid and mitigate potential catastrophes. Endosymbiosis, where AI becomes an integral part of human life, seems plausible given current trends, challenges, and policy constraints.

In this scenario, the subsequent steps may include (1) developing advanced neuroprosthetics and brain-computer interfaces for seamless AI integration into human cognition, (2) fostering AI-assisted decision-making systems that preserve human autonomy while leveraging AI’s computational prowess, and (3) implementing AI-driven augmentation technologies that enhance human physical capabilities, sensory perception, and communication abilities. Next, AI-enhanced humans may guide the emergence of autonomous AI agents with their own goals, ambitions, and corresponding utility functions. While similar scenarios have been extensively explored in science fiction and futuristic writings, we argue that these scenarios are becoming plausible evolutionary outcomes, falling into the same framework as a global adaptive response driven by the summed utility functions of all entities across the biosphere and noosphere – to survive and to develop new areas and niches for expansion. Furthermore, we propose employing concepts of complexity dynamic range and punctuated differentiation/cooperation principles to aid in modeling such adaptive responses in complex systems.

Taking inspiration from biological evolution, we can also apply adaptive changes and evolutionary principles to AI systems during integration, better aligning them with humanistic values and expectations. As we progress towards greater AI-human integration, considering the role of evolutionary trade-offs and constraints in shaping complexity in biological systems is important. This process may lead to new ecological niches, increased complexity, and the transformation of our civilization, which could be crucial for our long-term survival in the face of potential catastrophes.

## Abbreviations

ADHD: attention deficit hyperactivity disorder
AI: artificial intelligence
BCI: brain-computer interface
DL: deep learning
GAN: generative adversarial network
JAIC: Joint Artificial Intelligence Center
ML: machine learning
R&D: research and development

## References

[1] Vernadsky V.I. (2004). Biosphere and Noosphere. M.: Iris-Press, 2004. 576 p..

[2] de Chardin P.T. (1955). The Phenomenon of Man. New York: Harper, 1955. 319 p..

[3] Kurzweil R. (2005). The Singularity is Near: When Humans Transcend Biology. New York: Viking, 2005. 652 p..

[4] Tipler F.J. (1994). The Physics of Immortality: Modern Cosmology, God and the Resurrection of the Dead. New York: Doubleday, 1994. 560 p..

[5] Steffen W., Grinevald J., Crutzen P., McNeill J. (2011). Philosophical Transactions of the Royal Society A: Mathematical, Physical and Engineering Sciences..

[6] Crutzen P.J., Stoermer E.F. (2000). IGBP Newsletter..

[7] Zalasiewicz J., Williams M., Smith A., Barry T.L., Coe A.L., Bown P.R., Brenchley P., Cantrill D., Gale A., Gibbard P. (2008). GSA Today..

[8] Lewis S.L., Maslin M.A. (2015). Nature.

[9] Guo Z., Zhang L., Li Y. (2010). PLoS One..

[10] Sandom C., Faurby S., Sandel B., Svenning J.C. (2014). Proc. Royal Soc. B: Biol. Sci..

[11] Lambin E.F., Meyfroidt P. (2011). Proc. Natl. Acad. Sci. USA..

[12] Arthur W.B. (2009). The Nature of Technology: What It Is and How It Evolves. New York: Simon & Schuster Inc., 2009. 256 p..

[13] Mokyr J. (2009). The Enlightened Economy: an Economic History of Britain 1700–1850. Yale University Press, 2009. 564 p..

[14] Castells M. (2010). The Information Age: Economy, Society and Culture. 2nd ed. Vol. 1. The Rise of the Network Society. Chichester: Wiley–Blackwell, 2010. 594 p..

[15] Vernadsky V.I. (1991). Scientific Thought as a Planetary Phenomenon. M.: Nauka, 1991. 271 p..

[16] FAOSTAT.

[17] Andersson L., Purugganan M. (2022). Proc. Natl. Acad. Sci. USA..

[18] Barnosky A.D. (2008). Proc. Natl. Acad. Sci. USA..

[19] Margulis L. (1993). Symbiosis in Cell Evolution: Microbial Communities in the Archean and Proterozoic Eons. New York: W.H. Freeman, 1993. 448 p..

[20] Glazko V.I., Zybaylov B.L., Kosovsky Y.G., Glazko G.V., Glazko T.T. (2021). The Holocene..

[21] Mayr U. (2001). Psychol. Aging..

[22] Newman P. (2001). Water Sci. Technol..

[23] Barabási A.L., Albert R. (1999). Science..

[24] Schrödinger E. (1994). What is Life? The Physical Aspect of the Living Cell. Cambridge University Press, 1944. 92 p..

[25] Swenson R. (1989). Systems Research..

[26] Maynard Smith J., Szathmáry E. (1995). The Major Transitions in Evolution. Oxford University Press, 1995. 360 p..

[27] Morowitz H.J. (2002). The Emergence of Everything: How the World Became Complex. Oxford University Press, 2002. 224 p..

[28] Shannon C.E. (1948). The Bell System Technical Journal..

[29] Margulis L. (1970). Origin of eukaryotic cells. Yale University Press, 1970. 349 p..

[30] Canfield D.E. (2005). Annual Review of Earth and Planetary Sciences..

[31] Nicolelis M.A. (2001). Nature.

[32] Martin W., Müller M. (1998). Nature.

[33] Spang A., Saw J.H., Jørgensen S.L., Zaremba-Niedzwiedzka K., Martijn J., Lind A.E., van Eijk R., Schleper C., Guy L., Ettema T.J.G. (2015). Nature.

[34] Takemura M. (2001). Journal of Molecular Evolution..

[35] Bell P.J. (2001). Journal of Molecular Evolution..

[36] Wolpaw J.R., Wolpaw E.W. (2012). Brain Computer Interfaces. Principles and Practise Oxford University Press USA, 2012. 424 p.2012.

[37] Hochberg U., Degu A., Fait A., Rachmilevitch S. (2012). Physiol. Plant..

[38] Birbaumer N., Kübler A., Ghanayim N., Hinterberger T., Perelmouter J., Kaiser J., Iversen I., Kotchoubey B., Neumann N., Flor H. (2000). IEEE Trans Rehabil. Eng..

[39] Donoghue J.P. (2002). Nat. Neurosci..

[40] Lebedev M.A., Nicolelis M.A. (2006). Trends in Neurosciences..

[41] Birkenbihl C., Salimi Y., Domingo-Fernándéz D., Lovestone S., Fröhlich H., Hofmann-Apitius M. (2020). Japanese Alzheimer’s Disease Neuroimaging Initiative, the Alzheimer’s Disease Neuroimaging Initiative. Alzheimers Dement (N.Y.), AddNeuroMed consortium.

[42] Widge A.S., Ellard K.K., Paulk A.C., Basu I., Yousefi A., Zorowitz S., Gilmour A., Afzal A., Deckersbach T., Cash S.S. (2017). Exp. Neurol..

[43] Bardin L.D., King P., Maher C.G. (2017). Med. J..

[44] Lubar J.F. (1997). Appl Psychophysiol Biofeedback..

[45] Yang W., Yuste R. (2017). Nat. Methods..

[46] Diamond J. (2002). Nature.

[47] Larson G., Fuller D.Q. (2014). Annual Review of Ecology, Evolution, and Systematics..

[48] Belyaev D.K. (1979). J. Hered..

[49] Zeder M.A. (2012). Biodiversity in Agriculture: Domestication, Evolution, and Sustainability. Cambridge: Cambridge University Press,.

[50] Bostrom N. (2014). Superintelligence: Paths, Dangers, Strategies. Oxford University Press, 2014. 352 p..

[51] Eiben A.E., Smith J. (2015). Nature.

[52] Somashekhar S.P., Sepúlveda M.J., Puglielli S., Norden A.D., Shortliffe E.H., Rohit Kumar C., Rauthan A., Arun Kumar N., Patil P., Rhee K. (2018). Annals of Oncology..

[53] Choi Y.I., Chung J.W., Kim K.O., Kwon K.A., Kim Y.J., Park D.K., Ahn S.M., Park S.H., Sym S.J., Shin D.B. (2019). Can. J. Gastroenterol Hepatol..

[54] Holland J.H. (1975). Adaptation in Natural and Artificial Systems: An Introductory Analysis with Applications to Biology, Control, and Artificial Intelligence. Cambridge: MIT Press, 1975. 232 p..

[55] Mitchell M. (1996). An Introduction to Genetic Algorithms. Cambridge: MIT Press, 1996. 221 p..

[56] Floreano D., Keller L. (2010). PLoS Biology..

[57] Floreano D., Dürr P., Mattiussi C. (2008). Evolutionary Intelligence..

[58] Miikkulainen R., Liang J., Meyerson E., Rawal A., Fink D., Francon O., Raju B., Shahrzad H., Navruzyan A., Duffy N. (2017). Artificial Intelligence in the Age of Neural Networks and Brain Computing. Academic Press,.

[59] Lehman J., Clune J., Misevic D., Adami C., Altenberg L., Beaulieu J., Bentley P.J., Bernard S., Beslon G., Bryson D.M. (2020). Artificial Life..

[60] Stanley K.O., Miikkulainen R. (2002). Evolutionary Computation..

[61] Dawkins R. (1976). The selfish gene. New York, Oxford: Oxford University Press, 1976. 224 p..

[62] Chayanov A.V. (1989). Peasant Economy: Selected Works. M.: Ekonomika, 1989. 3491 p..

[63] Buss L.W. (1987). The Evolution of Individuality. Princeton University Press, 1987. 201 p..

[64] Carmina E., Longo A., Lobo R.A. (2003). Am. J. Obstet Gynecol..

[65] Luo L., O’Leary D.D. (2005). Annual Review of Neuroscience..

[66] Kahneman D., Klein G. (2009). American Psychologis..

[67] Goodfellow I.J., Pouget-Abadie J., Mirza M., Xu B., Warde-Farley D., Ozair S., Courville A., Bengio Y. (2014). Proceedings of the 27th International Conference on Neural Information Processing Systems,.

[68] Turkle S. (2011). Alone Together: Why We Expect More from Technology and Less from Each Other. Basic Books, 2011. 384 p..

[69] Damasio A. (2010). Self Comes to Mind: Constructing the Conscious Brain. Pantheon, 2010. 384 p..

[70] Dehaene S., Lau H., Kouider S. (2017). Science..

[71] Kandel E.R., Schwartz J.H., Jessell T.M., Siegelbaum S.A., Hudspeth A.J. (2013). Principles of Neural Science (5th edition). N.Y.: McGraw-Hill Medical, 2013. 1760 p..

[72] Daugherty P., Wilson H.J. (2018). Human + machine: Reimagining work in the age of AI. Boston, MA: Harvard Business Review Press, 2018. 264 p..

[73] Brynjolfsson E., McAfee A. (2014). The Second Machine age: Work, Progress, and Prosperity in a Time of Brilliant Technologies. New York: WW Norton & Company, 2014. 336 p..

[74] Tegmark M. (2017). Life 3.0: Being Human in the Age of Artificial Intelligence. New York: Knopf, 2017. 384 p..

[75] Sutton R.S., Barto A.G. (2018). Reinforcement learning: An introduction (2nd ed.). Cambridge: MIT Press, 2018. 552 p..

[76] Russell S.J. (2019). Human Compatible: Artificial Intelligence and the Problem of Control. Viking, 2019. 349 p..

[77] Bulliet R.W. (1990). The Camel and the Wheel. Columbia University Press, 1990. 327 p..

[78] Hewlett R.G., Anderson O.E. (1962). The New World, 1939– 1946. A History of the United States Atomic Energy Commission, Vol. 1. Pennsylvania, 1962. 766 p..

[79] Hafner K., Lyon M. (1996). Where Wizards Stay Up Late: The Origins of the Internet. Simon Schuster, 1996. 304 p..

[80] Scharre P. (2018). Army of None: Autonomous Weapons and the Future of War. New York: W.W. Norton Company, 2018. 448 p..

[81] Allen G., Kania E. (2017). China is using America’s own plan to dominate the future of artificial intelligence. Foreign Policy, 2017..

[82] (2018). JAIC. Department of Defense Joint Artificial Intelligence Center, 2018..

[83] (2017). State Council. New Generation Artificial Intelligence Development Plan. State Council of the People’s Republic of China, 2017..

[84] Horowitz M.C. (2018). Bulletin of the Atomic Scientists..

[85] Taddeo M., Floridi L. (2018). Nature.

[86] Crootof R. (2016). University of Pennsylvania Law Review..

[87] Nye J.S. (2017). International Security..

[88] (2017). Partnership on AI. Partnership on AI: About Us. 2017..

[89] Hanson R. (1988). The Great Filter: Are we almost past it? Online essay. 1998..

[90] Fermi E. (1950). Where is everybody? Unpublished conversation, 1950. 13 p..

[91] Webb S. (2002). If the universe is teeming with aliens... where is everybody?: Fifty solutions to the Fermi Paradox and the problem of extraterrestrial life. Springer Science & Business Media, 2002. 304 p..

[92] Bostrom N. (2002). Journal of Evolution and Technology..

[93] Ćirković M.M. (2008). Astrobiology..

[94] Yudkowsky E. (2008). Artificial intelligence as a positive and negative factor in global risk. In N. Bostrom & M. M. Ćirković (Eds.), Global Catastrophic Risks. Oxford University Press, 2008..

[95] Russell S., Dewey D., Tegmark M. (2015). AI Magazine..

[96] Dafoe A. (2018). AI Governance: A Research Agenda. Oxford, UK: Future of Humanity Institute, University of Oxford, 2018. 53 p..

[97] Muehlhauser L. (2015). Superintelligence and the future of governance: On prioritizing the control problem at the end of history. In B. Goertzel & T. Goertzel (Eds.), The End of the Beginning: Life, Society, and Economy on the Brink of the Singularity. Humanity Press, 2015. 738 p..

[98] Grace K., Salvatier J., Dafoe A., Zhang B., Evans O. (2018). Journal of Artificial Intelligence Research..

[99] Zubrin R. (1996). The Case for Mars: The plan to settle the red planet and why we must. The Free Press, 1996. 416 p..

[100] Musk E. (2017). New Space..

[101] Sagan C. (1994). Pale Blue Dot: A vision of the human future in space. Random House, 1994. 429 p..

[102] Bostrom N. (2003). Utilitas..

[103] Huntington S.P. (1996). The Clash of Civilizations and the Remaking of World Order. New York: Simon & Schuster, 1996. 368 p..

[104] Floridi L. (2018). Philosophy & Technology..

[105] Mittelstadt B.D., Allo P., Taddeo M., Wachter S., Floridi L. (2016). Big Data & Society..

